# Molecular detection and characterization of *Trichomonas* spp. in wild birds in the Brazilian Pantanal

**DOI:** 10.1590/S1984-29612025033

**Published:** 2025-06-16

**Authors:** Amanda Garcia Pereira, Sarah Raquel Jesus Santos Simões, Maitê Cardoso Coelho da Silva, Leticia Colovatti Mariano, Ana Cláudia Calchi, Amir Salvador Alabi Cordova, Tiago Valadares Ferreira, João Batista Pinho, Alan Fecchio, Ricardo Bassini-Silva, Ana Carolina Castro-Santiago, Jeffrey Bell, Rosangela Zacarias Machado, Marcos Rogério André, Karin Werther

**Affiliations:** 1 Serviço de Patologia de Animais Selvagens – SEPAS, Departamento de Patologia, Reprodução e Saúde Única, Faculdade de Ciências Agrárias e Veterinárias – FCAV, Universidade Estadual Paulista “Júlio de Mesquita Filho” – UNESP, Jaboticabal, SP, Brasil; 2 Vector-Borne Bioagents Laboratory – VBBL, Departamento de Patologia, Reprodução e Saúde Única, Faculdade de Ciências Agrárias e Veterinárias, Universidade Estadual Paulista “Júlio de Mesquita Filho” – UNESP, Jaboticabal, SP, Brasil; 3 Programa de Pós-Graduação em Ecologia e Conservação da Biodiversidade/Instituto de Biociências, Universidade Federal de Mato Grosso– UFMT, Cuiabá, MT, Brasil; 4 Department of Ornithology, Academy of Natural Sciences of Drexel University, Philadelphia, PA, USA; 5 Laboratório de Coleções Zoológicas, Instituto Butantan, São Paulo, SP, Brasil; 6 Departamento de Medicina Veterinária Preventiva e Saúde Animal, Faculdade de Medicina Veterinária e Zootecnia, Universidade de São Paulo – USP, São Paulo, SP, Brasil; 7 Department of Biology, University of North Dakota, Grand Forks, North Dakota, USA

**Keywords:** Avian parasites, wild birds, phylogenetic analysis, Protozoa, Trichomonas gallinae, Parasitas aviários, aves selvagens, análise filogenética, protozoários, Trichomonas gallinae

## Abstract

Members of the family Trichomonadidae, particularly *Trichomonas gallinae*, are globally distributed avian parasites that primarily infect birds from the orders Columbiformes, Falconiformes, and Strigiformes. Although infections in Passeriformes are often subclinical, clinical cases have been reported. Transmission occurs through direct contact or indirectly via contaminated food or water, enabling infection across various avian orders, including Anseriformes, Falconiformes, Galliformes, Gruiformes, Passeriformes, Piciformes, Psittaciformes, and Strigiformes. This study aimed to assess the occurrence and genetic diversity of *Trichomonas* spp. in 246 wild birds captured in Poconé, Mato Grosso, located in the Brazilian Pantanal. Oropharyngeal swab samples were collected in July 2022 from birds belonging to six different orders. Following DNA extraction, molecular detection was performed targeting the ITS1/5.8S/ITS2 ribosomal region. Of the total samples, 107 (43.5%) tested positive, with high prevalence in Passeriformes (40.8%), Cuculiformes (75%), and Columbiformes (63.2%). Phylogenetic analysis using Bayesian inference placed the 18 obtained sequences, representing five distinct haplotypes, into three separate clades of *T. gallinae*. In conclusion, *Trichomonas* DNA was detected in asymptomatic birds from three different avian orders. The high infection prevalence and haplotype sharing among species highlight the widespread distribution and potential transmission of *T. gallinae* among wild birds in the Brazilian Pantanal.

## Resumo

*Trichomonas* spp., especialmente *Trichomonas gallinae*, são parasitas aviários da família Trichomonadidae com distribuição global, afetando principalmente aves das ordens Columbiformes, Falconiformes e Strigiformes. Embora infecções em Passeriformes sejam frequentemente subclínicas, casos clínicos já foram documentados. A transmissão ocorre por contato direto ou indiretamente por meio de alimentos e água contaminados, permitindo a infecção de diversas ordens, como Anseriformes, Falconiformes, Galliformes, Gruiformes, Passeriformes, Piciformes, Psittaciformes e Strigiformes. Este estudo teve como objetivo avaliar a ocorrência e diversidade genética de *Trichomonas* spp. em 246 aves silvestres, capturadas em Poconé, Mato Grosso, região do Pantanal brasileiro. Amostras de *swab* orofaríngeo foram coletadas em julho de 2022 de aves pertencentes a seis ordens. Após a extração do DNA, realizaram-se testes moleculares com base na região ITS1/5.8S/ITS2. Do total, 107 (43,5%) amostras foram positivas, com alta prevalência em Passeriformes (40,8%), Cuculiformes (75%) e Columbiformes (63,2%). A análise filogenética por inferência Bayesiana posicionou as 18 sequências obtidas (cinco haplótipos distintos) em três clados de *T. gallinae*. Em conclusão, DNA de *Trichomonas* foi detectado em aves assintomáticas de três ordens distintas, indicando alta prevalência e compartilhamento de haplótipos entre diferentes espécies no Pantanal brasileiro.

## Introduction

Avian *Trichomonas gallinae* infections can lead to severe lesions in the upper digestive tract, including inflammation of the esophagus (esophagitis) and the crop (ingluvitis). These lesions often result in the accumulation of caseous necrotic material, which can obstruct the esophagus and make food ingestion difficult. Consequently, infected birds may experience weight loss, weakness, and, in severe cases, death. Studies indicate that lesion severity varies widely, ranging from small superficial ulcers to extensive masses that significantly impair feeding. Early detection and appropriate management strategies are essential to minimizing disease impacts in both wild and domestic bird populations. (Amin et al., 2014).

Pigeons (*Columba livia*) are particularly susceptible hosts and can act as reservoirs of the infection, facilitating transmission among other bird species (Forzán et al., 2010). In passerines, the infection can result in high morbidity and mortality, severely affecting population health and biodiversity, especially in threatened species (Quillfeldt et al., 2018). Additionally, trichomoniasis in wild birds can interfere with conservation programs, as the disease may be a limiting factor in the recovery of endangered populations.

In pigeons, the infection often manifests through necrotic lesions in the oral mucosa and pharynx, leading to feeding difficulties, coughing, and labored breathing, along with lethargy and rapid weight loss (Forrester & Foster, 2008). In contrast, passerines exhibit more subtle clinical signs, such as less pronounced oral lesions, mild lethargy, difficulty singing, and compromised reproductive health, which may result in later diagnoses (Martínez et al., 2023).

Previous studies have highlighted the importance of monitoring and sanitary management for containing the infection in wild and domestic birds, emphasizing the need for an integrated approach to avian health (Quillfeldt et al., 2018). The present study aimed to investigate the occurrence and genetic diversity of trichomonads in different species of wild birds to explore the possible association between the parasite haplotypes and infected bird species.

## Material and Methods

### Study area and captured birds

**Figure 1:**
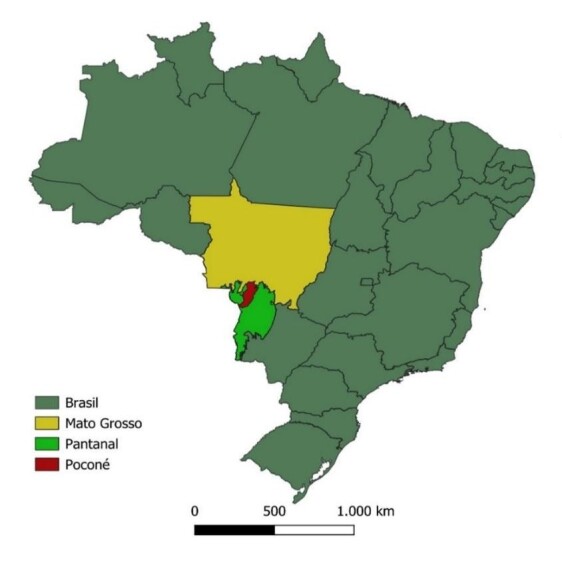
Geographic location of the study area, highlighting Poconé (Microrregion of the Pantanal) in the state of Mato Grosso, central-western Brazil.

Birds were captured in the state of Mato Grosso, in the municipality of Poconé (Figure 1), latitude -16.2571, longitude -56.6246 and altitude 153 meters, in July 2022, over a period of 10 days. A total of 246 birds (Table 1) were captured using two mist nets, and one swab of the oropharyngeal mucosa were collected from each bird. All captured birds had their nails painted with red nail polish for possible identification, and in cases where birds were captured again, they were released.

**Table 1:** Number of bird species captured in Poconé, Mato Grosso, Brazil, and their respective taxonomic orders.

**Number of specimens**	**Order**	**Number of specimens sampled according to avian species**
2	Accipitriformes	2 *Rupornis magnirostris*
19	Columbiformes	8 *Leptotila verreauxi*, 9 *Columbina talpacoti* and 2 *Columbina squammata*
4	Cuculiformes	3 *Crotophaga ani*, 1 *Coccycua minuta*
2	Galbuliformes	1 *Momotus momota* and 1 *Galbula cyanicollis*
225	Passeriformes	13 *Turdus amaurochalinus*, 6 *Synallaxis albilora*, 8 *Furnarius rufus*, 2 *Cercomacra melanaria*, 2 *Certhiaxis cinnamomeus*, 58 *Ramphocelus carbo*, 5 *Taraba major*, 3 *Pseudoseisura unirufa*, 11 *Eucometis penicillata*, 5 *Pipra fasciicauda*, 1 *Furnarius leucopus*, 1 *Pitangus sulphuratus*, 3 *Cnemotriccus fuscatus*, 3 *Hemitriccus margaritaceiventer*,, 6 *Myiothlypis flaveola*, 5 *Poecilotriccus latirostris*, 1 *Hypocnemoides maculicauda*, 1 *Synallaxis albescens*, 1 *Saltator coerulescens*, 1 *Dendroplex picus*, 5 *Cranioleuca vulpina*, 2 *Icterus pyrrhopterus*, 4 *Casiornis rufus*, 2 *Fluvicola albiventer*, 2 *Thraupis palmarum*, 2 *Sporophila leucoptera*, 2 *Camptostoma obsoletum*, 2 *Myiophobus fasciatus*, 2 *Pheugopedius genibarbis*, 2 *Arremon flavirostris*, 3 *Turdus leucomelas*, 2 *Paroaria capitata*, 1 *Elaenia cristata*, 1 *Sporophila caerulescens*, 3 *Thamnophilus pelzelni*, 39 *Volatinia jacarina*, 3 *Myiarchus ferox*, 2 *Machetornis rixosa*, 1 *Inezia inornata*, 2 *Coryphospingus cucullatus*, 4 *Sicalis flaveola*, 1 *Tolmomyias sulphurescens*, 1 *Campylorhamphus trochilirostris* and 1 *Oryzoborus angolensis*
4	Piciformes	3 *Veniliornis passerinus*, and 1 *Picumnus albosquamatu*,

This swab was stored in a microtube containing buffered saline solution (PBS; pH 7.2) and kept in liquid nitrogen, then transferred to a -80 °C freezer until DNA extraction and molecular assays for trichomonads were performed. All procedures were authorized by the CEUA (n° 732/21) and SISBIO (n° 77538-1).

In our study, it was not possible to apply the fresh wet mount method for the detection of motile *Trichomonas* due to an operational constraint: the impracticality of transporting a microscope to the Pantanal without compromising its calibration. Although this method is simple and effective for the direct identification of motile protozoa, it requires a properly calibrated microscope in optimal conditions for field analysis. Transporting the equipment to a remote region like the Pantanal could result in loss of calibration, making it difficult to obtain clear and reliable images. Additionally, factors such as humidity, vibrations, and adverse environmental conditions could further affect the accuracy of microscopic observations. For these reasons, we opted to use alternative methods to ensure the integrity of the samples for subsequent laboratory analysis.

### DNA extraction and PCR for endogenous avian *β-actin* gene

For DNA extraction, swab samples were heated in a dry bath at 40 °C for 15 minutes. After this procedure, the excess PBS was removed, leaving approximately 300 microliters in the microtube. With the swab still in the microtube, proteinase K and lysis buffer were added according to the instructions of the commercial kit used (Biopur Mini Spin Plus, MoBius Life Science®, Paraná, Brazil). The swab was removed only before transferring the contents to the centrifugation column, strictly following the remaining steps of the manufacturer's protocol.

To eliminate false negative results due to the presence of inhibitors, DNA extractions were subjected to conventional PCR aimed at amplifying the endogenous avian *β-actin* gene. For this, the primers β-actin-F (5’-ATCTCGTCTTGTTTTATGCG-3’) and β-actin-R (5’-TATCCGTAAGGATCTGTATG-3’) were used (van Borm et al., 2007). The cycling protocol and the sequences of the primers used are described in Table 2. Sterilized ultra-pure water was used as a negative control in all PCR assays.

**Table 2:** Primer sequences and thermal cycling conditions used in conventional PCR assays for *T. gallinae* detection.

**MOLECULAR MARKER**	**PRIMER SEQUENCES**		**THERMAL SEQUENCES**	**REFERENCES**	**FRAGMENT SIZE**
**ENDOGENOUS GENE**					
β actinF	5’-CCTCATGAAGATCCTGACAGA3’		95 °C for 5 min, 95 °C for 30s, 54 °C for 30s, 72 °C for 1min, 72 °C for 5 min	van Borm et al. (2007)	700 bp
β actinR	5’-TCTCCTGCTCYAAYTCCA-3’				
**ITS1/5.8S/ITS2**					
**TRF1**	(5’ -TGCTTCAGCTCAGCGGGTCTTCC-3’)		94 °C for 2 min, 94 °C for 20s, 66 °C for 20s, 72 °C for 30s, 72 °C for 5 min	Felleisen (1997)	369 bp
**TRF2**	(5’ - CGGTAGGTGAACCTGCCGTTGG-3’)				
**SSUrRNA**					
**Hm-Long-f**	(5′-AGGAAGCACACTATGGTCATAG-3′)		95 °C for 15 min, 94 °C for 30s, 55 °C for 1 min, 72 °C for 2 min, 72 °C for 10 min	Ganas et al. (2014)	1500 bp
**Hm-Long-r**	(5′-CGTTACCTTGTTACGACTTCTCCTT-3′)				
***Fe-hydrogenase***					
**TrichhydFOR**		(5’- GTTTGGGATGGCCTCAGAAT-3’)	95 °C for 10 min, 96 °C for 5s, 53 °C for 3s, 68 °C for 15s. A final extension of 72 °C for 10s. Samples held at 10 °C	Lawson et al. (2011)	1000 bp
**TrichhydREV**		(5’- AGCCGAAGATGTTGTCGAAT-3’)			

### Molecular screening and characterization for *Trichomonas* spp.

Samples positive for the endogenous avian *β-actin* gene were screened for *Trichomonas* spp. through a conventional PCR based on the ITS1/5.8S rRNA/ITS2 regions (Felleisen, 1997). DNA from *Trichomonas gallinae* (DNA samples of birds from Jaboticabal) was used as a positive control in the PCR assays for *Trichomonas* spp. Samples that tested positive in the PCR assay for the ITS1/5.8S rRNA/ITS2 regions were characterized through PCR assays targeting the 18S rRNA (Ganas et al., 2014) and *Fe-hydrogenase* (Lawson et al., 2011) genes. The amplified products from the cPCR assays were subjected to horizontal electrophoresis on a 1.0% agarose gel stained with Ethidium Bromide (0.5 µL/mL) in TBE running buffer. The results were visualized and analyzed using a UV transilluminator (ChemiDoc MP Imaging System, BIO RAD®).

### Sequencing, BLASTn analysis and phylogenetic analyses

The obtained amplicons were purified using the Wizard® SV Gel and PCR Clean-Up System (Promega, Fitchburg, WI, USA), following the manufacturer's recommendations. Sequencing of the amplified products was performed using the chain termination method with dideoxynucleotides (Sanger et al., 1977), conducted on the ABI PRISM 3700 DNA Analyzer (Applied Biosystems®) at the Biological Resource Center and Genomic Biology (CREBIO - FCAV - UNESP).

The Phred-Phrap version 23 program (Ewing & Green, 1998) was used to construct consensus sequences from the previously described molecular markers, analyzing both the forward and reverse strands sequenced from the same sample, ensuring a minimum quality value of 20 for each nucleotide to determine the nucleotide sequence. The BLAST program (Altschul et al., 1990) was used to compare the obtained sequences with those deposited in GenBank (Benson et al., 2005).

Sequences were aligned with other homologous sequences of the same gene retrieved from the database (GenBank), using Clustal/W software (Thompson et al., 1994) via Bioedit v. 7.0.5.3 (Hall, 1999). Alignments were transformed into Nexus format using the Alignment Transformation Environment website (Glez-Peña et al., 2010). Bayesian analysis was conducted using MrBayes 3.2.2 on XSEDE (Ronquist & Huelsenbeck, 2003) via the CIPRES portal (Miller et al., 2015). 

Bayesian analysis was performed with 10^6 generations and the number of substitution classes varied according to the evolutionary model found for each data set. The evolutionary model was determined using jModelTest 2 software (Darriba et al., 2012). The editing of phylogenetic trees and rooting (via outgroups) was performed using Treegraph 2.0.56-381 beta software (Stöver & Müller, 2010).

### Diversity analysis and haplotype network

To calculate nucleotide diversity (π), polymorphism level (haplotype diversity – [dh]), number of haplotypes (h), and mean number of nucleotide differences (K) between the obtained sequences, DnaSP v5 software was used (Librado & Rozas, 2009). Additionally, the sequences were subjected to TCSnetwork analysis (Bandelt et al., 1999) using the Population Analysis with Reticulate Trees (popART) software (Leigh & Bryant, 2015). This analysis was used to verify haplotype diversity based on different gene targets among the sampled wild birds.

## Results

Of the 246 DNA samples extracted from oropharyngeal swabs, the presence of the endogenous avian *β-actin* gene was detected in 226 samples (91.86%), collected from 50 species (Table 3).

**Table 3:** The number of DNA samples from each avian species that underwent PCR amplification for the *β-actin* gene, and the number of PCR positive samples.

**Species**	**Examined**	**Positive**
*R. magnirostris*	2	2
*L. verreauxi*	8	7
*C. talpacoti*	9	9
*C. squammata*	2	2
*C. ani*	3	3
*C. minuta*	1	1
*M. momota*	1	1
*T. amaurochalinus*	13	9
*S. albilora*	6	5
*F. rufus*	8	7
*C. melanaria*	2	1
*C. cinnamomeus*	2	1
*R. carbo*	58	54
*T. major*	5	4
*E. penicillata*	11	10
*P*. *fasciicauda*	5	4
*F. leucopus*	1	1
*P*. *sulphuratus*	1	1
*C*. *fuscatus*	3	3
*H*. *margaritaceiventer*	3	3
*M. flaveola*	6	5
*P. latirostris*	5	5
*H*. *maculicauda*	1	1
*S. albescens*	1	1
*S. coerulescens*	1	1
*D. picus*	1	1
*C*. *vulpine*	5	2
*I. pyrrhopterus*	2	1
*C. rufus*	4	4
*F. albiventer*	2	2
*T. palmarum*	2	2
*S*. *leucoptera*	2	2
*C. obsoletum*	2	2
*M. fasciatus*	2	2
*P. genibarbis*	2	2
*A. flavirostris*	2	2
*T. leucomelas*	3	3
*P. capitata*	2	2
*E*. *cristata*	1	1
*S. caerulescens*	1	1
*T. pelzelni*	3	3
*V. jacarina*	39	39
*M. ferox*	3	2
*M. rixosa*	2	1
*I. inornata*	2	1
*C. cucullatus*	2	2
*S. flaveola*	4	4
*V*. *passerines*	2	1
*T. sulphurescens*	1	1
*C. trochilirostris*	1	1
*O. angolensis*	1	1

Of these, 107 (43.49%) were positive for *Trichomonas* spp. based on the ITS1/5.8S/ITS2 region: 92/225 (40,88%) Passeriformes, 3/4 (75%) Cuculiformes, and 12/19 (63.15%) Columbiformes (Table 4).

**Table 4:** The number of DNA samples from each avian species that underwent PCR amplification for the *ITS1/5.8S/ITS2* region of *T. gallinae*, and the number of PCR positive samples.

**Species**	**Examined**	**Positive**
*S. albilora*	5	1
*F. rufus*	7	3
*C. melanaria*	1	1
*C. cinnamomeus*	1	1
*R. carbo*	54	26
*T. major*	4	2
*E. penicillata*	10	7
*P. fasciicauda*	4	3
*C. fuscatus*	3	2
*H. margaritaceiventer*	3	1
*M. flaveola*	5	2
*P. latirostris*	5	3
*S. albescens*	1	1
*S. coerulescens*	1	1
*I. pyrrhopterus*	1	1
*C. rufus*	4	1
*F. albiventer*	2	1
*T. palmarum*	2	1
*S. leucoptera*	2	1
*C. obsoletum*	2	1
*M. fasciatus*	2	2
*P. genibarbis*	2	1
*A. flavirostris*	2	1
*T. leucomelas*	3	2
*E. cristata*	1	1
*S. caerulescens*	1	1
*T. pelzelni*	3	2
*V. jacarina*	39	17
*C. cucullatus*	2	2
*S. flaveola*	4	2
*T. sulphurescens*	1	1
*C. ani*	3	3
*L. verreauxi*	7	5
*C. talpacoti*	9	5
*C. squammata*	2	2

The strongest and most unique bands were chosen for sequencing, making it possible to obtain 18 good sequences and these were deposited in Genbank with accession numbers PQ740247-PQ740264. All 107 samples were negative in the PCR assays based on the *Fe-hydrogenase* and 18S rRNA genes.

The sequences obtained in this study had an identity ranging from 99.18% to 100% with *Trichomonas* sp. sequences detected in *Ramphastos dicolorus* (ON000429) from Brazil and in columbiforms from USA (EU215361 and EU215360).

**Figure 2:**
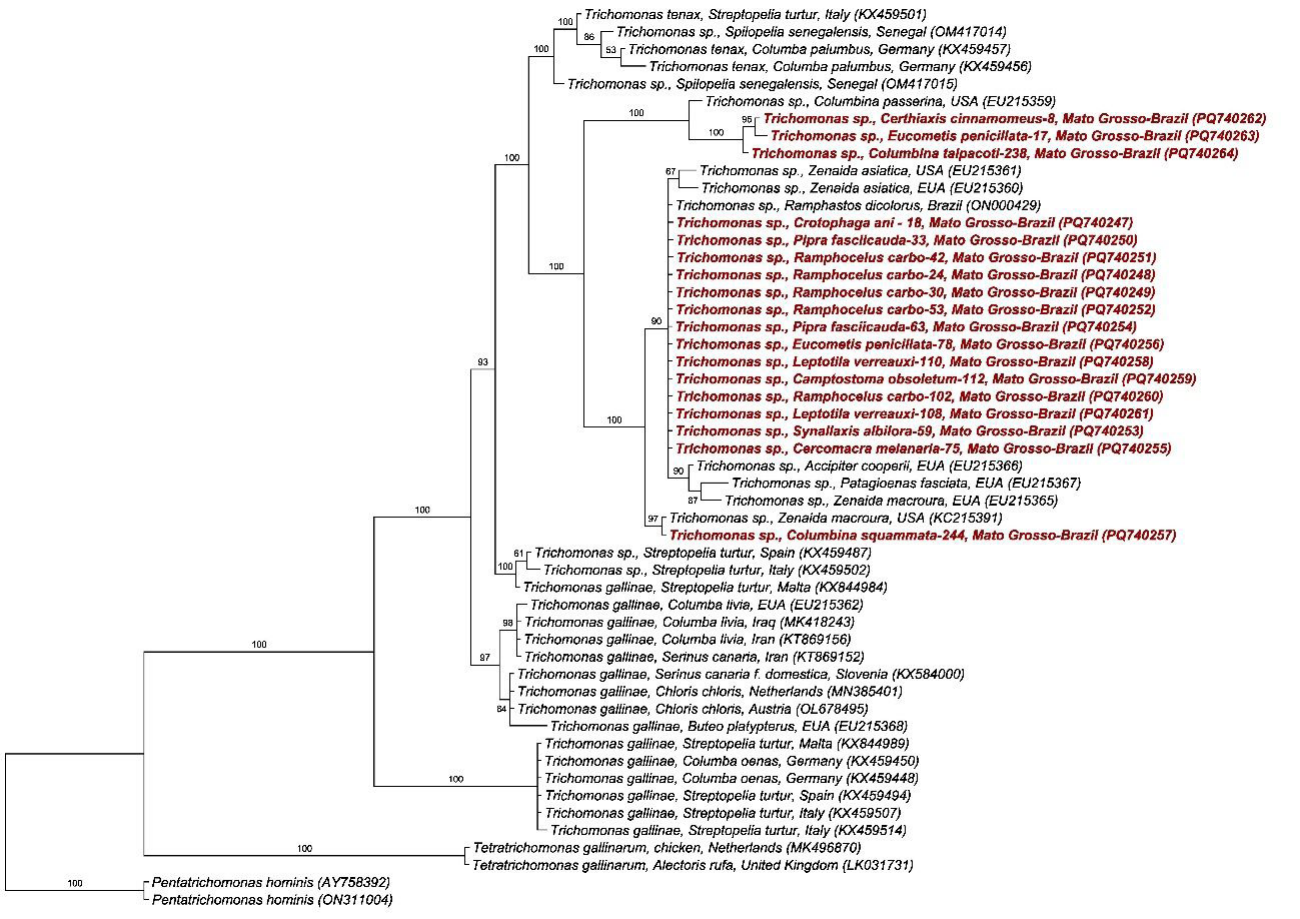
Bayesian inference phylogenetic tree of *Trichomonas* spp. based on a 318 bp alignment of the ITS1/5.8S/ITS2 region, using the TIM2+I evolutionary model. *Pentatrichomonas hominis* sequences were used as the outgroup. Numbers above internodes indicate posterior probability support values.

Bayesian inference phylogenetic reconstruction implementing the TIM2+I evolutionary model, based on a 318 bp alignment of the ITS1/5.8S/ITS2 region of *Trichomonas* spp., positioned the sequences obtained in this study into three distinct clades of *Trichomonas* spp. (Figure 2). One sequence detected in *C. squammata* grouped with a sequence of *Trichomonas* sp. detected in *Zenaida macroura* (USA). The majority of the sequences (detected in *R. carbo*, *C. ani*, *P. fasciicauda*, *E. penicillata*, *L. verreauxi*, *S. albilora*, *C. melanaria*, and *C. obsoletum*) were positioned in a clade along with sequences detected in *Ramphastos dicolorus* in Brazil and *Zenaida asiatica* in the USA. Finally, 3 sequences (*C*. *cinnamomeus*, *E. penicillata*, and *C. talpacoti*) were positioned in the clade containing a sequence detected in *Columbina passerina* in the USA.

A total of 18 sequences from the ITS1-5.8S-ITS2 regions were analyzed, yielding 18 different haplotypes, of which five contained sequences detected in birds sampled in this study: haplotypes #2, #3, #4, #6, and #9. Haplotype diversity (h) was 0.892 ± 0.031 and nucleotide diversity (π) was 0.08476 ± 0.01110, with 92 variable sites, an average number of nucleotide differences (K) of 23.22364, and 34.1% G+C content.

**Figure 3:**
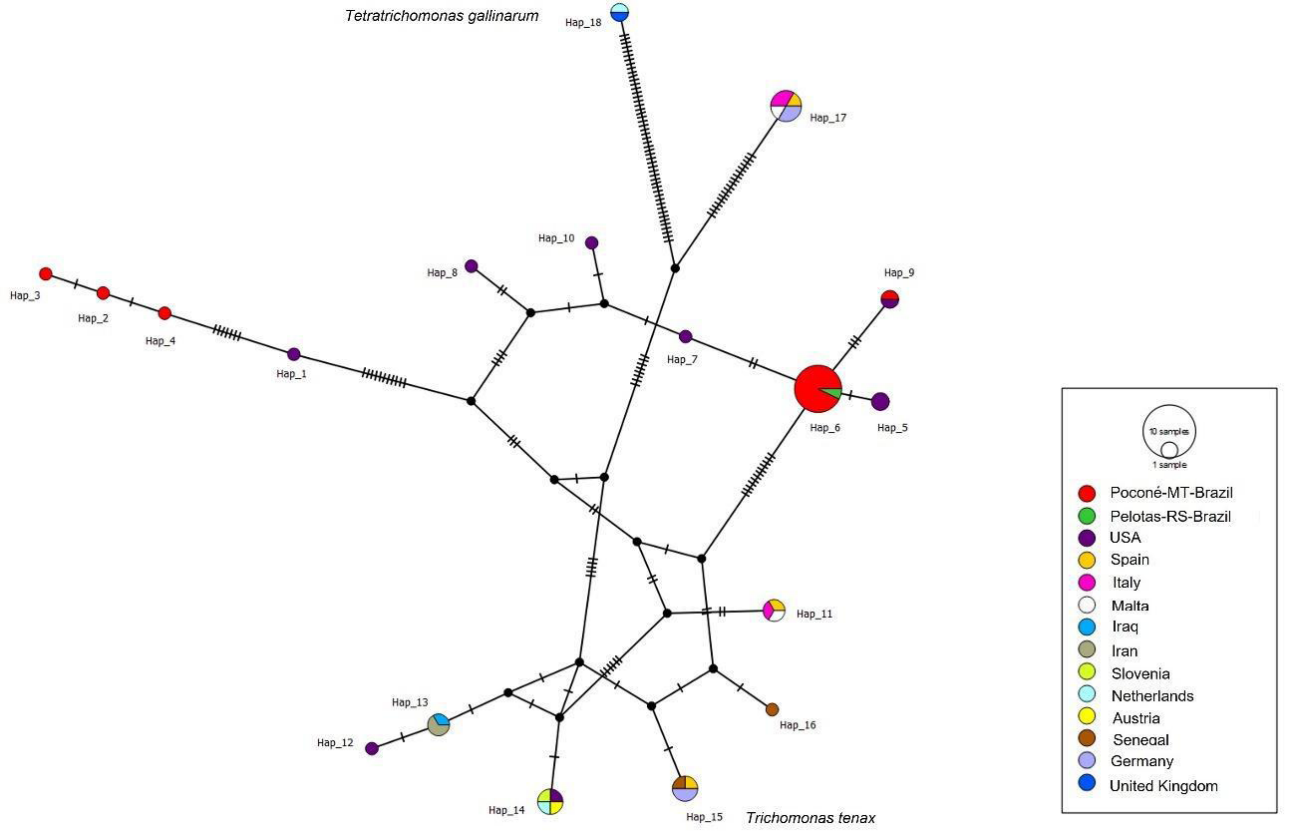
Haplotype network based on the ITS1/5.8S/ITS2 regions of *Trichomonas* spp. Colors indicate the geographic origins of the analyzed sequences. Black points represent median vectors (hypothetical sequences not included in the dataset), and vertical black lines indicate the number of mutational events between haplotypes.

Haplotype #6, contained 14 sequences from the Brazilian Pantanal (detected in 1 *C. ani*, 2 *P. fasciicauda*, 4 *R. carbo*, 1 *E. penicillata*, 2 *L. verreauxi*, 1 *C. obsoletum*, 1 *S. albilora*,1 *C. melanaria*) and 1 sequence detected in Pelotas from *Ramphastos dicolorus*. Haplotype #6, was separated from haplotypes #7 and #9 by 2 mutational events, from haplotype #5 by 1 mutational event, and from a median vector by several mutational events. Haplotype #9 included 1 sequence from the Pantanal (*C. squammata*) and 1 sequence detected in the USA (*Z. macroura*). Finally, haplotypes #2, #3, and #4 were formed by unique sequences detected in *C. cinnamomeus*, *C. talpacoti*, and *E. penicillata* in Poconé, MT, respectively (Figure 3).

## Discussion

This study revealed a high prevalence of *Trichomonas* spp. in wild birds from the Brazilian Pantanal, with different infection rates among the sampled orders: Columbiformes (12/19, 63.15%), Passeriformes (92/225, 40.88%), and Cuculiformes (3/4, 75%). These findings corroborate with previous studies highlighting the susceptibility of Columbiformes to this parasite, often associated with pigeons (*C. livia*), which act as important reservoirs in urban and rural environments (Martins et al., 2007; Lawson et al., 2011). The high prevalence observed in Cuculiformes, although limited by the small sample size, suggests that these omnivorous birds may also be important hosts, requiring further investigations to confirm their epidemiological role.

The literature highlights that the sharing of food and water is an important transmission route of *T. gallinae*, especially in anthropized environments (Forzán et al., 2010). In our study, the high prevalence in Passeriformes, particularly in species such as *R. carbo*, which inhabit urban areas, may indicate the relevance of these environments in the maintenance and dissemination of the parasite. Similar studies suggest that the proximity of wild and domestic birds in urban areas increases the risk of transmission (Gerhold et al., 2013).

Several studies have reported the presence of *T. gallinae* in passerines. Quillfeldt et al. (2018) documented infection in 34 out of 132 (25.75%) passerines sampled in Hesse, Germany, between 2015 and 2017, highlighting the potential risk posed by this parasite. In Iran, Arabkhazaeli et al. (2020) collected 349 passerines, of which 60 (17.2%) tested positive. Their findings suggest that passerines may serve as important hosts, contributing to the epidemiology of *T. gallinae*. In the present study, 40.88% prevalence was observed in passerines, which is considerably higher than reported by Arabkhazaeli et al. (2020) or Quillfeldt et al. (2018). These results indicate that the sampled passerines exhibit a high level of infection, possibly related to ecological or regional factors. In addition, the different diagnostic methodologies and oligonucleotide primers used may have influenced the results obtained in different studies.

In Columbiformes, there are several reports of *T. gallinae*, with studies demonstrating high infection rates. For instance, Dunn et al. (2023) observed that all 149 Columbiformes sampled in West Africa (Senegal) during two winters tested positive for *T. gallinae*. Tuska-Szalay et al. (2022) reported that 72/99 (73%) pigeons (*C. livia*) collected in Hungary and Romania, tested positive for *T. gallinae*. Only a small percentage of the infected birds exhibited clinical signs, whereas most were asymptomatic, reinforcing their role as silent carriers of this parasite. In the present study, the infection rate in Columbiformes was 63.15%, a value consistent with previous results. This high prevalence demonstrates the importance of Columbiformes as natural reservoirs of *T. gallinae* and their potential impact on the parasite's dissemination. Trichomoniasis is recognized as a potential threat to endangered species, especially endemic Passeriformes (Quillfeldt et al., 2018). Continuous monitoring in regions such as the Pantanal is essential to prevent negative impacts on local biodiversity.

Genotypic analysis revealed significant diversity among positive samples, with five distinct haplotypes of *T. gallinae*. This diversity aligns with studies emphasizing the parasite's broad distribution and ecological plasticity (Albeshr & Alrefaei, 2019). A notable finding was the detection of haplotypes shared between Columbiformes and Passeriformes, indicating that the shared consumption of fruits and seeds may be an important mechanism for interspecies transmission (Chi et al., 2013). Additionally, the detection of similar haplotypes across different geographic regions, such as between *C. squammata* in Brazil and *Zenaida macroura* in the USA, suggests a high dispersal capacity of these parasites which may explain the global connectivity of *T. gallinae* haplotypes.

Despite the high prevalence detected, no birds exhibited clinical signs characteristic of trichomonosis. The absence of lesions may be attributed to the presence of less virulent strains or possible acquired immunity. Previous studies, such as those by Lawson et al. (2011) and Chi et al. (2013), also reported subclinical infections in wild birds, suggesting that the clinical manifestation of the disease depends on both the strain's virulence and the host's immune status.

The lack of *Fe-hydrogenase* gene amplification in all positive samples limited more detailed genotypic analyses. Lawson et al. (2011) and Alrefaei et al. (2019) highlighted that this gene can provide higher resolution for discriminating *T. gallinae* strains. However, technical difficulties in amplifying this gene are common and have been previously reported (Chi et al., 2013).

The failure to amplify the *Fe-hydrogenase* and 18S rRNA genes in all positive samples reinforces the limitation of using only the ITS1/5.8S rRNA/ITS2 region for species identification. Previous studies (e.g., Lawson et al., 2011; Chi et al., 2013) highlight that additional gene targets can provide higher resolution for distinguishing *Trichomonas* species. However, amplification difficulties for these genes are known and may be influenced by factors such as DNA quality, primer specificity, or genetic variability among *Trichomonas* spp. This methodological limitation implies that the detected *Trichomonas* could belong to various species, from *Tritrichomonas* spp. to *Tetratrichomonas* spp., and not exclusively *T. gallinae.*

Finally, the higher prevalence observed in bird species associated with urban areas, such as *C. talpacoti* and *R. carbo*, underscores the need for continuous monitoring in anthropized environments. These findings highlight the importance of integrated strategies for sanitary management and conservation to mitigate risks to local biodiversity and threatened bird populations. The presence of *C. livia* in urban areas, frequently infected with *T. gallinae*, may act as a significant source of infection for wild birds (Gerhold et al., 2013). These findings highlight the importance of integrated strategies for sanitary management and conservation, considering the potential risks to local biodiversity and threatened bird populations. Although all captured birds underwent oropharyngeal examination with no clinical signs of trichomoniasis observed, continuous monitoring remains essential due to the possibility of asymptomatic transmission in urban environments.

## Conclusion

This study provided new records of the occurrence and genetic diversity of *T. gallinae* in wild birds from the Brazilian Pantanal, highlighting the parasite's widespread distribution across different avian orders and host species with different life histories. The high occurrence in Columbiformes, Passeriformes, and Cuculiformes reinforces the ecological role of these birds as potential hosts of *T. gallinae*. In anthropized environments infected individuals that may act as significant reservoirs. Genotypic analysis revealed notable diversity of *T. gallinae*, including haplotypes shared among phylogenetically unrelated bird species and geographic regions, suggesting a common transmission mechanism mediated by the shared consumption of water sources, fruits and seeds. Although no birds exhibited clinical signs of the disease, the molecular identification of the parasite in different species underscores the need for continuous monitoring, particularly in areas where domestic and wild birds interact.

## Data Availability

Data will be made available on request.
